# Immune Memory to Sudan Virus: Comparison between Two Separate Disease Outbreaks

**DOI:** 10.3390/v7010037

**Published:** 2015-01-06

**Authors:** Ariel Sobarzo, Yael Eskira, Andrew S. Herbert, Ana I. Kuehne, Spencer W. Stonier, David E. Ochayon, Shlomit Fedida-Metula, Steven Balinandi, Yaara Kislev, Neta Tali, Eli C. Lewis, Julius Julian Lutwama, John M. Dye, Victoria Yavelsky, Leslie Lobel

**Affiliations:** 1 Department of Microbiology, Immunology and Genetics, Faculty of Health Sciences, Ben-Gurion University of the Negev, Beer-Sheva 8410501, Israel; E-Mails: tautau.ariel@gmail.com (A.S.); askiray@gmail.com (Y.E.); fedida.metula@gmail.com (S.F.-M.); k.yaarula@gmail.com (Y.K.); netatali20@gmail.com (N.T.); yavelsky@bgu.ac.il (V.Y.); 2 Virology Division—U.S. Army Medical Research Institute of Infectious Diseases, 1425 Porter St., Fort Detrick, Frederick, MD 21701, USA; E-Mails: anderw.s.herbert.ctr@mail.mil (A.S.H.); ana.kuehne@us.army.mil (A.I.K.); spencer.w.stonier2.ctr@mail.mil (S.W.S.); john.m.dye1.civ@mail.mil (J.M.D.); 3 Department of Clinical Biochemistry and Pharmacology, Faculty of Health Sciences, Ben-Gurion University of the Negev, Beer-Sheva 8410501, Israel; E-Mails: davidochayon@gmail.com (D.E.O.); lewis@bgu.ac.il (E.C.L.); 4 Department of Arbovirology, Emerging and Re-emerging Infection Uganda Virus Research Institute, Entebbe P.O Box 49, Uganda; E-Mails: hnp6@ug.cdc.ug (S.B.); jjlutwama03@yahoo.com (J.J.L.)

**Keywords:** ebolavirus, human survivors, memory immunity, cross-reactivity

## Abstract

Recovery from ebolavirus infection in humans is associated with the development of both cell-mediated and humoral immune responses. According to recent studies, individuals that did not survive infection with ebolaviruses appear to have lacked a robust adaptive immune response and the expression of several early innate response markers. However, a comprehensive protective immune profile has yet to be described. Here, we examine cellular memory immune responses among survivors of two separate Ebolavirus outbreaks (EVDs) due to Sudan virus (SUDV) infection in Uganda—Gulu 2000–2001 and Kibaale 2012. Freshly collected blood samples were stimulated with inactivated SUDV, as well as with recombinant SUDV or Ebola virus (EBOV) GP (GP_1–649_). In addition, ELISA and plaque reduction neutralization assays were performed to determine anti-SUDV IgG titers and neutralization capacity. Cytokine expression was measured in whole blood cultures in response to SUDV and SUDV GP stimulation in both survivor pools, demonstrating recall responses that indicate immune memory. Cytokine responses between groups were similar but had distinct differences. Neutralizing, SUDV-specific IgG activity against irradiated SUDV and SUDV recombinant proteins were detected in both survivor cohorts. Furthermore, humoral and cell-mediated crossreactivity to EBOV and EBOV recombinant GP_1–649_ was observed in both cohorts. In conclusion, immune responses in both groups of survivors demonstrate persistent recognition of relevant antigens, albeit larger cohorts are required in order to reach greater statistical significance. The differing cytokine responses between Gulu and Kibaale outbreak survivors suggests that each outbreak may not yield identical memory responses and promotes the merits of studying the immune responses among outbreaks of the same virus. Finally, our demonstration of cross-reactive immune recognition suggests that there is potential for developing cross-protective vaccines for ebolaviruses.

## 1. Introduction

Ebolaviruses are members of the *Filoviridae* family, which contains a single-stranded, negative-sense RNA genome encoding seven genes [1]. These genes encode a nucleoprotein (NP), viral proteins VP35 and VP40, a glycoprotein (GP), viral proteins VP30 and VP24 and the viral RNA-dependent RNA polymerase (L) [2,3]. Among the different proteins expressed by ebolaviruses, the surface glycoprotein, GP, plays a central role in viral attachment, entry, cytotoxicity, as well as induces both cellular and humoral immune responses, and activates pro-inflammatory pathways [3,4].

In July 2012, an Ebola virus outbreak (EVD) occurred in the Kibaale district, Uganda. EVD can be caused by four distinct ebolaviruses: Bundibugyo, Ebola, Sudan, and Taï Forest virus. Laboratory tests during the outbreak confirmed Sudan virus (SUDV) infection in eleven patients resulting in four deaths. This ebolavirus, first discovered in 1976, reemerged in the Gulu district of Uganda in 2000–2001 and was responsible for 425 confirmed infections and 224 deaths [5,6].

During the last decade, various studies that examine the pathogenesis of ebolavirus infection in humans have indicated that recovery is largely dependent on the development of both cell-mediated immunity and an effective humoral immune response [4,7]. Ebolavirus infection triggers the release of cytokines and chemokines, including interleukin (IL)-1β, IL-6, IL-8, IL-15 and interferon-γ (IFNγ) [8]. In addition, evidence from studies that examined survivors and asymptomatic cases, demonstrated the presence of significant levels of virus-specific IgM and IgG titers associated with a temporary, early and strong inflammatory response [9,10,11,12]. However, a comprehensive protective immune profile has yet to be described.

Here, we investigate the presence of cellular memory immune responses in human survivors of SUDV from two different EVD outbreaks in Uganda using a whole blood cytokine stimulation assay. Samples were collected on-site from five of the seven survivors of the 2012 EVD outbreak due to SUDV infection that occurred in Kibaale, and were compared to six survivors of the 2000 EVD disease outbreak due to SUDV infection in the Gulu district, which were previously shown to contain raised humoral and cellular immunity [12]. To this end, freshly collected heparinized blood samples were added to preloaded reaction tubes containing specific stimulators; post-stimulation plasma supernatants were then isolated and analyzed. Specific stimulation conditions included inactivated SUDV, recombinant SUDV GP_1–649_ and recombinant Ebola virus (EBOV) GP_1–649_. Post-stimulation analytes included IL-1β, IL-2, IL-5, IL-10, IFNγ and TNFα. Additionally, SUDV-specific IgG levels and SUDV neutralization capacity were also assessed.

## 2. Materials and Methods

### 2.1. Study Design

Subjects included confirmed survivors from the SUDV-caused EVD outbreaks of 2000–2001 and 2012 in Gulu and Kibaale districts, Uganda, respectively [5,13], and healthy local community members of each district that were not infected. Study participants were unrelated (except two isolated samples from Kibaale) and, according to outbreak medical records, infection progression and prognosis did not necessarily exhibit genetic predisposition.

### 2.2. Ethics Statement

The study was approved by the Helsinki committees of the Uganda Virus Research Institute in Entebbe, Uganda (reference number GC/127/13/01/15), Soroka Hospital, Beer-sheva, Israel (protocol number 0263-13-SOR) and the Ugandan National Council for Science and Technology (UNCST) (registration number HS1332). A written informed consent was translated for and signed by each subject; a personal health questionnaire was completed for each subject at the time of blood collection.

### 2.3. Sample Collection

Whole blood samples were obtained from individuals by routine antecubital venipuncture. Samples were directly aspirated into sterile vacutainers containing freeze-dried sodium heparin (final heparin concentration 14.3 units/mL, Becton Dickinson, Franklin Lakes, NJ, USA) and kept at 4 °C until assayed within a uniform timeframe.

### 2.4. Antigens and Stimulants

Stimulation assay antigens included irradiated, sucrose gradient purified, SUDV-gul (Sudan virus /H. sapiens-tc/UGA/Gulu-808892) and purified recombinant (His-tagged) SUDV-gul or EBOV (Ebola virus/H. sapiens-tc/COD/1995/Kikwit) GP viral polypeptide, residues 1 to 649 (GP_1–649_) [14]. A lectin from *Phaseolus vulgaris* Leucoagglutinin, PHA-L, (Sigma-Aldrich, Israel) was used as positive control for cell stimulation. For ELISA assays, irradiated SUDV-gul, purified recombinant (His-tagged) SUDV-gul GP_1–649_, and total 293T cell lysate that expressed a given recombinant SUDV-gul protein (NP, VP30, VP35 and VP40) were used as the capture antigens. Construction of the recombinant SUDV viral gene expression vectors and production of irradiated SUDV and EBOV have been described previously [15]. Cell lysates from cultures not expressing recombinant viral protein were used as control. Irradiated, sucrose gradient purified, EBOV and purified recombinant (His-tagged) EBOV GP_1–649_ were also used as capture antigens for cross reactivity-assessing ELISA.

### 2.5. Internal Control Sera

Internal human control sera for ELISA were previously described [14]. Positive controls for the detection of SUDV GP_1–649_ and EBOV GP_1–649_ included murine monoclonal antibodies 3C10 and 6D8 that target the SUDV and EBOV GP, respectively [16].

### 2.6. Specific IgG Detection Assays

The levels of circulating anti-SUDV, anti-SUDV recombinant viral proteins, anti-EBOV and anti-EBOV GP_1–649_ antibodies were determined by chemiluminescence ELISA, as previously described [14] with minor modifications. A 100 μL volume of total cell lysate expressing SUDV Flag tag, recombinant viral protein (antigen) or cell lysate (mock antigen) at a concentration of 15 μg/well, or 2 μg/mL of purified SUDV or EBOV GP_1–649,_ or whole virus SUDV or EBOV antigens in phosphate buffered saline (PBS) without Mg^+2^ and Ca^+2^, pH 7.4, was passively adsorbed onto ELISA plates (MaxiSorp, Nunc™, Thermo Fisher Scientific Inc., Waltham, MA, USA) overnight in a humidified chamber at 4 °C. After incubation, plates were washed three times with 0.1% (v/v) Tween-20 (Sigma-Aldrich Israel Ltd., Rehovot, Israel) in PBS; the same washing procedure followed each subsequent stage of the assay. Plates were then blocked with 10% (w/v) skim milk (Merck, Darmstadt, Germany) in PBS. After incubation at 37 °C for 1 h, plates were washed and triplicate 100 μL volumes of each tested serum and controls at 1:400 dilution were added to wells containing the recombinant viral proteins and mock antigens. Further controls, including an anti-FLAG murine monoclonal antibody, goat anti-SUDV serum and murine anti-SUDV and EBOV GP_1–649_ monoclonal antibodies were added at a dilution of 1:1000, 1:3000 and 1:1000, respectively. After incubation for 1 h at 37 °C, plates were washed, and a volume of 100 μL goat anti-human IgG HRP conjugate (1:10,000, Santa Cruz Biotechnology, Dallas, TX, USA) was added to wells. For murine anti-Flag, anti-GP_1–649_, and goat anti-SUDV antiserum controls, a volume of 100 μL goat anti-mouse and donkey anti-goat IgG HRP conjugate (1:10,000, Santa Cruz Biotechnology) were added to the wells, respectively. After incubation, plates were washed, and 40 μL of oxidizing reagent (H_2_O_2_) and enhanced luminal reagent solutions (NEL105 chemiluminescence reagent kit) were loaded at a 1:1 ratio. Optical absorbance was measured using a standard luminometer (Infinite F200, TECAN, Männedorf, Switzerland).

### 2.7. Normalization of Raw Data and Selection of Cut-off Values

Calculation of signal to noise (S/N) values, for anti-SUDV recombinant proteins NP, VP30, VP35, and VP40 specific IgG, was performed as previously described [14]. The cutoff value for IgG positive immunoreactivity was determined with a control set of negative sera and ten-fold stratified cross-validation analysis [17]. For the purified SUDV, EBOV and GP_1–649_ proteins, raw ELISA data were converted to percent positivity (PP) of a high internal control antibody. Calculation of PP values, as well as the cutoff value, was performed as previously described [18]. Normalization of cytokine expression levels in whole blood stimulation assays was performed by removing background (unstimulated expression) for each respective stimulated sample.

### 2.8. Plaque Reduction Neutralization Test

Plaque reduction neutralization assays (PRNT_50_) were performed as previously described [19]. Six ten-fold serial dilutions of serum were mixed with 100 plaque-forming units of SUDV or EBOV at 37 °C for 1 h in the presence or absence of 5% guinea pig complement (Cedarlane, Burlington, NC, USA). Following incubation, 125 μL of the reaction mix was loaded onto Vero cell monolayers in 96 well plates (Costar, Cambridge, MA, USA). Cells were overlaid with agarose-based medium and a second overlay containing 5% neutral red was added 8 days later. Plates were then incubated at 37 °C, with 5% CO_2_ over-night, and plaques were counted the following day. Neutralization titers were determined to be the last dilution of serum that reduced the number of plaques by 50% compared with control wells. Plaque reduction neutralization assays were performed in the BSL-4 lab of USAMRIID (Fort Detrick, Frederick, MD, USA).

### 2.9. Whole Blood Stimulation from Ebolavirus Survivors and Healthy Volunteers

Whole blood stimulation was performed as previously described [20] with minor modifications. Heparinized venous blood obtained from SUDV survivors and healthy volunteers was aliquoted into 12 × 75 mm snap-cap polypropylene tubes under sterile conditions. Each blood sample from both survivor and control subjects was diluted 1:4 in RPMI-1640 supplemented with 5% FCS. Irradiated SUDV antigen (10 μg/mL), SUDV GP_1–649_ (50 μg/mL), or EBOV GP_1–649_ (50 μg/mL), were added to individual aliquots (1.0 mL final vol.), in duplicates, and the cultures were incubated at 37 °C/5% CO_2_ for 22 h. PHA-L (10 μg/mL) was used as a positive control for cell activation, negative control consisted of a non-stimulated culture. Following incubation, culture supernatants were aspirated, transferred to new 1.5 mL tubes and frozen at –70 °C until further processing.

### 2.10. Cytokine Detection Using Q-Plex™ ELISA-Based Chemiluminescent Assay

Levels of human cytokines were detected using Q-Plex technology (Quansys Biosciences, Logan, UT, USA) according to manufacturer's instructions. Briefly, 30 μL of each sample (duplicate) was diluted 1:2 in diluent buffer, and a total volume of 50 μL of diluted sample and control standards was added to a 96-well v-bottom binding plate. Plates were then incubated for 1 h on a shaker at room temperature in the dark. Following incubation, plates were washed and a volume of 50 μL of detection mix was added to each well, and incubated for 1 h. Next, plates were washed and a volume of 50 μL streptavidin HRP was added to each well and incubated for additional 15 min. Finally, plates were washed and a volume of 50 μL substrate mix was added to each well. Readouts were obtained with a Quansys Imager (Quansys Biosciences, Logan, UT, USA) and results analyzed using the Q-View Software program (Quansys Biosciences).

### 2.11. Statistical Analysis

Statistical analyses were performed using GraphPad Prism software 6.01 (GraphPad Software, Inc., La Jolla, CA, USA). Differences in cytokine values between study groups were assessed by analysis of variants (ANOVA) and Wilcoxon rank sum test; *p*-values represent 2-sided *p*-values (0.05), and *p*-values < 0.05 were considered statistically significant.

## 3. Results

### 3.1. Cohorts and Blood Samples

Whole blood samples were obtained from a total of twenty donors. Of these, eleven were collected from survivors of the 2000–2001 and 2012 EVD outbreaks in Gulu (*n* = 6 infected and *n* = 4 non-infected) and Kibaale (*n* = 5 infected and *n* = 5 non-infected), respectively. Samples were collected from the Gulu cohort and Kibaale cohort approximately 12 years and 1 year post infection, respectively. Samples from healthy personnel (Soroka Hospital, Beer-Sheva, Israel) were used to compare baseline immune response levels (*n* = 3, data served for background reduction and are not shown). All subjects reported lack of autoimmune diseases, cancer and past hospitalizations that are unrelated to EVD (Tables S1 and S2). Survivors were hospitalized for 10–30 days during the outbreak and reported uniform treatment and post-recovery symptoms during and following SUDV infection (not shown).

### 3.2. Humoral Response Profiles against SUDV Proteins

Kibaale survivors had mixed immune-reactivity against SUDV-derived recombinant viral proteins: all reacted against VP40, four against NP, GP_1–649_ and SUDV-gul whole virus, three against VP30 and two against VP35 (Table 1).

Serum collected from all five survivors demonstrated neutralization activity against live SUDV. As expected, no neutralization or immune reactivity was observed in the non-infected control group.

Samples from survivors of the Gulu 2000–20001 EVD outbreak were previously analyzed [12] and were retested as comparison against Kibaale survivors. All survivors in this study displayed positive immune-reactivity against SUDV, as well as to recombinant viral proteins NP, VP30 and GP_1–649_ (Table 2).

Four individuals displayed serum reactivity to VP40, and one against VP35. Neutralization assay results demonstrated positive SUDV neutralization in five samples. No neutralization or reactivity to inactivated virus or recombinant proteins was observed in the non-infected control group.

**Table 1:** Serology and neutralization of Kibaale survivors. Summary of ELISA immunoreactivity, and PRNT_50_, in human survivor sera from Kibaale (S1–S5) and non-infected controls (N1–N5), against the different viral recombinant proteins of SUDV and the whole viral antigen.

	*Serology*						*PRNT_50_*
	*VP30*	*VP35*	*VP40*	*NP*	*GP_1–649_* ^1^	*SUDV Whole Ag*	*SUDV*
**S-1**	+++	-	++	+++	+++	+++	+++
**S-2**	++	-	+++	+++	+++	+++	+++
**S-3**	-	+	++	++	++	++	++
**S-4**	-	-	+++	-	-	-	+
**S-5**	++	+	++	+++	+++	+++	+++
**N-1**	-	-	-	-	-	-	-
**N-2**	-	-	-	-	-	-	-
**N-3**	-	-	-	-	-	-	-
**N-4**	-	-	-	-	-	-	-
**N-5**	-	-	-	-	-	-	-

¹ A purified recombinant protein containing the 649 amino terminal amino acid of SUDV GP without the trans-membrane domain. S—Ebola survivors, N—Non-infected control ELISA and neutralization assays results were divided into low (+), medium (++) and strong (+++) immunoreactivity or neutralization capability. For ELISA: (+)—(Lower than 2x cut off value), (++)—(2x cut off value – 4x cut off value), (+++)—(Greater than 4x cut off value). For neutralization assay (PRNT50): (+)—(Neutralizes at 1:20 dilution), (++)—(Neutralizes at 1:40 dilution), (+++)—(Neutralizes at greater than 1:80 dilution).

**Table 2:** Serology and neutralization of Gulu survivors. Summary of ELISA immunoreactivity, and PRNT_50_, in human survivor sera from Gulu (S1–S6), and non-infected controls (N1–N4), against the different viral recombinant proteins of SUDV and the whole viral antigen.

	*Serology*						*PRNT_50_*
	*VP30*	*VP35*	*VP40*	*NP*	*GP_1–649_* ^1^	*SUDV Whole Ag*	*SUDV*
**S-1**	++	-	+	+++	+++	+++	+++
**S-2**	+	-	+	+++	+++	+++	+
**S-3**	+	+	-	+++	+++	++	+++
**S-4**	+++	-	-	+	+++	+++	+++
**S-5**	++	-	+	+++	+++	++	-
**S-6**	+++	-	+++	+++	+++	+++	++
**N-1**	-	-	-	-	-	-	-
**N-2**	-	-	-	-	-	-	-
**N-3**	-	-	-	-	-	-	-
**N-4**	-	-	-	-	-	-	-

¹ A purified recombinant protein containing the 649 amino terminal amino acid of SUDV GP without the trans-membrane domain. S—Ebola survivors, N—Non-infected control ELISA and neutralization assays results were divided into low (+), medium (++) and strong (+++) immunoreactivity or neutralization capability. For ELISA: (+)—(Lower than 2x cut off value), (++)—(2x cut off value—4x cut off value), (+++)—(Greater than 4x cut off value). For neutralization assay (PRNT50): (+)—(Neutralizes at 1:20 dilution), (++)—(Neutralizes at 1:40 dilution), (+++)—(Neutralizes at greater than 1:80 dilution).

### 3.3. SUDV-Induced Cytokine Levels in Whole Blood Stimulation Assay

To identify markers of cell-mediated immune responses to SUDV, cytokine levels were determined following whole blood stimulation using SUDV, recombinant SUDV GP_1–649_, or recombinant EBOV GP_1–649._ PHA-L stimulation was used as a positive control for immune cell responses and non-stimulated samples served as baseline controls. SUDV survivors from Kibaale and Gulu exhibited equivalent baseline levels of cytokines compared to healthy non-infected controls (data not shown). PHA-L stimulation control exhibited a positive response in all tested groups, including healthy non-infected controls (data not shown).

**Figure 1:**
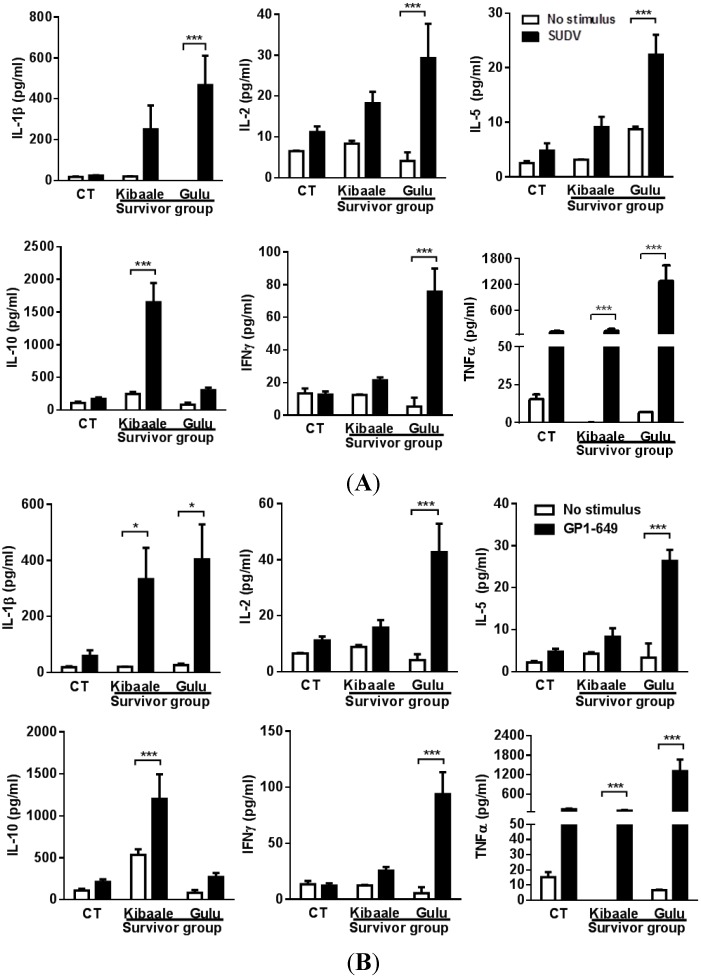
Cytokine levels comparison between SUDV-Gul whole antigen (**A**); and GP_1–649_ (**B**) stimulation and unstimulated whole blood from Kibaale (1-year) and Gulu (12-year) ebolavirus survivors and non-infected controls (CT). Non-infected controls of Kibaale and Gulu are presented as average results since there were no significant changes between the two groups (Data not shown). Cytokine levels were measured in the plasma supernatants of samples following whole blood stimulation and resting state. Mean ± SEM, * *p* < 0.05.

Stimulation of whole blood samples with SUDV or SUDV GP elicited cytokine secretion that was markedly elevated relative to resting cultures as well as uninfected controls (Figure 1).

One year after the outbreak, Kibaale survivors demonstrated a significant increase in IL-1β, IL-10, and TNFα, and elevated expression levels of IL-2, IL-5 and IFNγ. Cytokine levels in the 12-year post-outbreak Gulu survivors demonstrated a stronger response than that observed in Kibaale survivors with a significant increase in IL-1β, IL-2, IL-5, IFNγ and TNFα levels, and elevated expression levels of IL-10. No significant change was observed in non-infected control group (CT).

**Figure 2:**
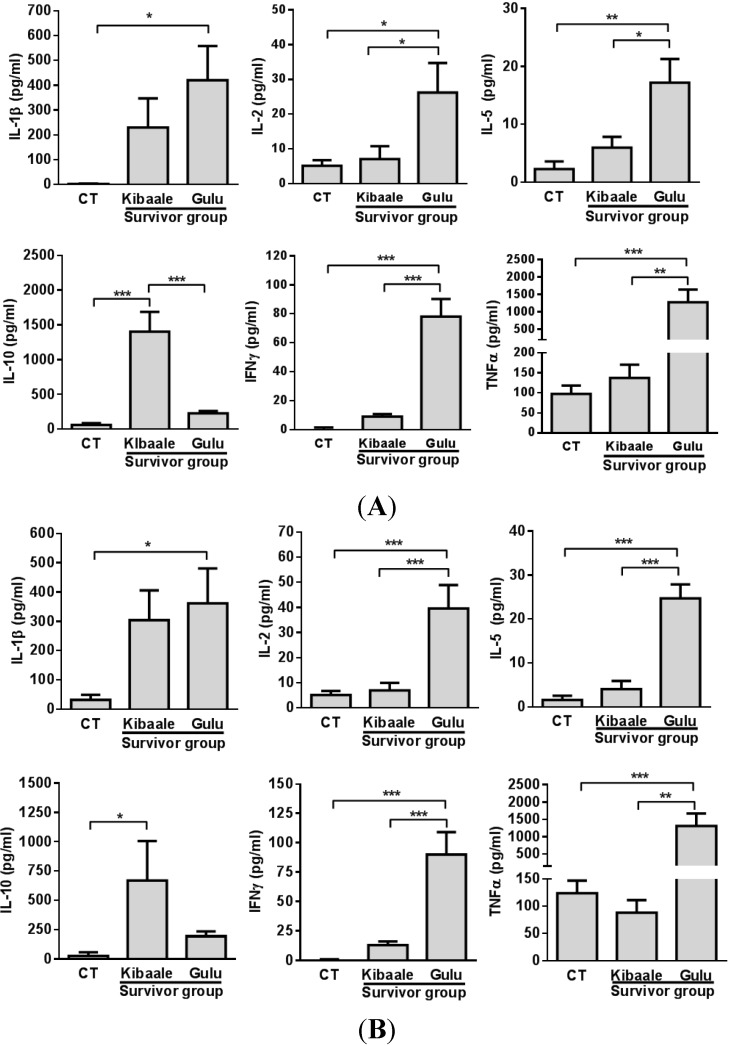
Cytokine levels following SUDV/Gul whole antigen (**A**) and GP_1–649_ (**B**) stimulation of whole blood from Kibaale (1-year) and Gulu (12-year) ebolavirus survivors and non-infected controls (CT). Cytokine levels were measured in the plasma supernatants of samples following whole blood stimulation. Normalization of cytokine expression levels was performed by reducing the background (unstimulated value) of each individual stimulated (SUDV/Gul whole antigen or GP_1–649_) sample. Non-infected controls of Kibaale and Gulu are presented as average results since there were no significant changes between the two groups of controls (Data not shown). Mean ± SEM, * *p* < 0.05.

Comparison of cytokine levels between SUDV Kibaale and Gulu survivors and non-infected controls (Figure 2), displayed a significant high cytokine response in samples of Gulu survivors compared to those of Kibaale and non-infected control groups following both SUDV and SUDV GP_1–649_ stimulation, with the exception of IL-10 expression levels.

### 3.4. Humoral, Cell-Mediated and Neutralization Cross Reactivity to EBOV GP_1–649_ in SUDV Survivors

In the Kibaale group, three individuals exhibited various degrees of cross-serological reactivity towards EBOV GP_1–649_ (Table 3) with two of those individuals showing cross reactivity towards EBOV whole virus. Results of survivors from Gulu group displayed a similar degree of crossreactivity to EBOV; three out of six individuals demonstrated cross reactivity towards EBOV GP_1–649_, and one displayed positive cross reactivity towards EBOV whole virus. Uninfected control serum exhibited no reactivity to EBOV or EBOV GP (Table 3).

**Table 3:** Serology and neutralization for EBOV cross-reactivity. Summary of ELISA immunoreactivity, and PRNT_50_, in human survivor sera from Kibaale (1-year) and Gulu (12-year) outbreaks, and non-infected controls, against the viral recombinant protein GP (GP_1–649_) of EBOV and the whole viral antigen.

Kibaale Group				Gulu Group			
	*EBOV Whole Ag*	*EBOV GP_1–649_*	*PRNT_50_*		*EBOV Whole Ag*	*EBOV GP_1–649_*	*PRNT_50_*
**S-1**	-	+	-	**S-1**	-	+++	-
**S-2**	+++	+++	-	**S-2**	-	-	-
**S-3**	-	-	-	**S-3**	-	+	-
**S-4**	-	-	-	**S-4**	-	-	-
**S-5**	+	++	-	**S-5**	-	-	-
**N-1**	-	-	-	**S-6**	++	++	-
**N-2**	-	-	-	**N-1**	-	-	-
**N-3**	-	-	-	**N-2**	-	-	-
**N-4**	-	-	-	**N-3**	-	-	-
**N-5**	-	-	-	**N-4**	-	-	-

S—Ebola survivors, N—Non-infected control ELISA results were divided into low (+), medium (++) and strong (+++) immunoreactivity: (+)—(Lower than 2x cut off value), (++)—(2x cut off value—4x cut off value), (+++)—(Greater than 4x cut off value). For neutralization assay (PRNT50): (+)—(Neutralizes at 1:20 dilution), (++)—(Neutralizes at 1:40 dilution), (+++)—(Neutralizes at greater than 1:80 dilution).

**Figure 3:**
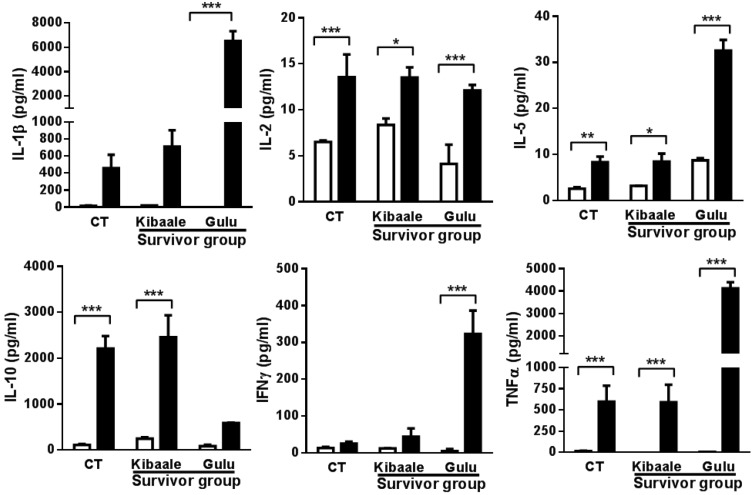
Cytokine levels comparison between EBOV GP_1–649_ stimulation and unstimulated whole blood from Kibaale (1-year) and Gulu (12-year) ebolavirus survivors and non-infected controls (CT). Cytokine levels were measured in the plasma supernatants of samples following whole blood stimulation. Non-infected controls of Kibaale and Gulu are presented as average results since there were no significant changes between the two groups of controls (Data not shown). Mean ± SEM, * *p* < 0.05.

*In vitro* whole blood assays were performed using EBOV GP_1–649_ to determine if SUDV survivors had cross-reactive memory cellular immunity with EBOV proteins. As demonstrated in Figure 3, highly inducible cytokines were detected in survivors and controls. Significant elevation in IL-1β, IL-2, IL-5, IFNγ and TNFα was observed in the Gulu cohort in response to EBOV GP_1–649_ stimulation.

**Figure 4:**
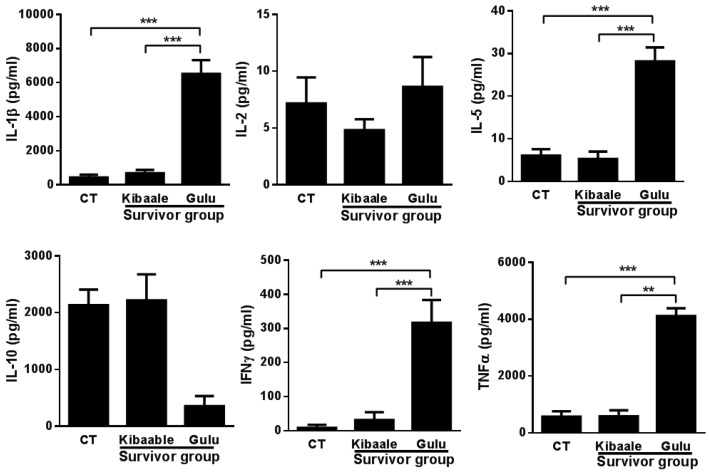
Cytokine levels following EBOV GP_1–649_ stimulation of whole blood from Kibaale (1-year) and Gulu (12-year) ebolavirus survivors and non-infected controls (CT). Cytokine levels were measured in the plasma supernatants of samples following whole blood stimulation. Normalization of cytokine expression levels was performed by reducing the background (unstimulated value) of each individual stimulated (EBOV GP_1–649_) sample. Non-infected controls of Kibaale and Gulu are presented as average results since there were no significant changes between the two groups (Data not shown). Mean ± SEM, * *p* < 0.05.

Significant elevation of IL-2, IL-5, IL-10 and TNFα expression levels was observed in the Kibaale survivor group and non-infected controls. Comparison between SUDV survivor groups and non-infected controls (Figure 4) demonstrated a significantly higher cytokine cross reactive response mainly in samples from the Gulu group of survivors.

## 4. Discussion

Filoviruses cause a severe hemorrhagic fever in humans resulting in a progressive and overwhelming disease [3]. Work in nonhuman primates and other smaller animal models demonstrates that adaptive immunity contributes to protection against ebolavirus infection [21,22] and that this immune protection is associated with the development of both cellular and humoral immunity [23,24,25,26,27].

In the work described herein, we present results of a study designed to investigate and compare markers of cellular and humoral memory immune responses in two distinct SUDV survivor cohorts. Assessing these parameters is essential for understanding immune parameters associated with protection against ebolavirus infection and to facilitate the development of effective vaccines and therapeutics. Using samples collected from SUDV survivors of the Kibaale outbreak in 2012, and the Gulu outbreak in 2000–2001 [12], the following parameters were compared: IgG immunoreactivity, neutralization capacity and cytokine expression levels following *in vitro* whole blood stimulation. The data were also compared to samples from non-infected individuals in both districts and to healthy off-site volunteers. The results of our study demonstrate that significant differences in cytokine expression levels and profile exist between SUDV survivor cohorts groups and non-infected controls. The immune responses in SUDV survivors from Kibaale were analyzed one year after the outbreak, whereas the Gulu outbreak survivors’ responses were determined 11 years after the outbreak. Despite the fact that the Gulu outbreak occurred over a decade prior to the outbreak in Kibaale, it is striking that Gulu survivors have more robust memory immune responses, as measured by cytokine production. Whether or not these differences can be attributed to varying environmental factors or supportive care given during the outbreak remains a point of speculation. Some cytokines, such as TNFα, IL-2, IFNγ and IL-5 are associated with T cell responses and therefore suggest T cell memory responses, but some cytokines associated with innate, pro-inflammatory immune responses were also detected. This is perhaps explained by a potential ability of the antigens used in this assay to stimulate innate immune cells, or could be a nonspecific activation event due to T cell cytokine production. Though this was not a focus in these assays, it would be intriguing if the recombinantly produced SUDV GP was inherently immuno-stimulatory. Nonetheless, the presence of T cell-associated cytokines vouches for an antigen-specific memory immune response.

Interestingly, the whole blood stimulation data reveals a significantly higher level of IL-10 expression in the Kibaale outbreak survivors. Although it remains speculative, this finding may suggest anti-inflammatory immunity among Kibaale outbreak survivors in contrast to those from Gulu, or may also reflect distinct time periods from infection.

The *in vitro* stimulation assay, while providing a clear picture of cellular responses, has several limitations. Whole blood inducible expression levels during stimulation were measured during one time point from the addition of stimulants to cells, and activation was performed using the SUDV/Gul isolate. Some cytokines require specific time frames for optimal peak detection, and might also display variation in response to particular stimulants. However, the undertaking of such an in-depth study under local conditions was not feasible at the point of sample collection; the maximal magnitude of sample replicates and added control conditions was reached in the present study design. Although the use of SUDV/Gul for activation during our assay limits the comparison between the two groups of survivors, it is unlikely that antigen isolate variation would be responsible for the significant difference in cytokine responses as genomic analysis between Gulu and Kibaale isolates reveals 99% sequence identity [5], and the humoral immune response presented in this work demonstrates robust recognition in both groups of survivors.

Humoral and cell-mediated cross reactivity against EBOV GP_1–649_ and EBOV demonstrated that SUDV survivor sera from both cohorts, regardless of the time post infection, reacted against EBOV whole virus and EBOV GP_1–649_. However, while survivor sera demonstrated positive recognition according to an ELISA, both Gulu and Kibaale groups lacked cross-neutralization capacity against EBOV. *In vitro* whole blood stimulation assays using EBOV GP_1–649_ demonstrated positive cross reactivity mainly in the Gulu cohort as compared to the Kibaale cohort. These high cross reactive profiles may reflect a stronger cellular immunity developed over the years. However, the elevated cytokine cross-stimulation profile could also result from differences in genetic background, geographic location, or the limited number of samples tested in the present work. Nevertheless, the overall humoral- and cell-mediated cross reactivity results identified in the present study suggest that a spectrum of immunity persists in long-recovered Sudan virus or EVD survivors, which is broader than may have been previously perceived.

The results of our study also have implications for vaccine development. SUDV survivors appeared to have developed a strong IgG immune response against the viral protein GP, and this viral protein evoked production of high cytokine expression levels following whole blood stimulation. These results are in agreement with previous studies [3,28,29], supporting the fact that activation of humoral and cellular neutralizing immunity could be achieved using vaccines based on the viral GP alone.

Overall, the data presented in this study is essential for providing further insights regarding the nature of the native human humoral and cellular immune response following EBOV infection. Such data will facilitate the development of optimized vaccines and immune therapeutics for filovirus infections.

## Supplementary Files

Supplementary File 1

## References

[1] Kuhn J.H. (2008). Filoviruses. A compendium of 40 years of epidemiological, clinical, and laboratory studies. Arch. Virol. Suppl..

[2] Ascenzi P., Bocedi A., Heptonstall J., Capobianchi M.R., di Caro A., Mastrangelo E., Bolognesi M., Ippolito G. (2008). Ebolavirus and marburgvirus: Insight the filoviridae family. Mol. Aspects Med..

[3] Feldmann H., Geisbert T.W. (2011). Ebola haemorrhagic fever. Lancet.

[4] Dolnik O., Kolesnikova L., Becker S. (2008). Filoviruses: Interactions with the host cell. Cell. Mol. Life Sci..

[5] Albarino C.G., Shoemaker T., Khristova M.L., Wamala J.F., Muyembe J.J., Balinandi S., Tumusiime A., Campbell S., Cannon D., Gibbons A. (2013). Genomic analysis of filoviruses associated with four viral hemorrhagic fever outbreaks in uganda and the democratic republic of the congo in 2012. Virology.

[6] (2001). Outbreak of Ebola Hemorrhagic Fever—Uganda, August 2000–January 2001. Can. Commun. Dis. Rep..

[7] Mohamadzadeh M., Chen L., Schmaljohn A.L. (2007). How ebola and marburg viruses battle the immune system. Nat. Rev. Immunol..

[8] Kondratowicz A.S., Maury W.J. (2012). Ebolavirus: A brief review of novel therapeutic targets. Future Microbiol..

[9] Gupta M., Goldsmith C.S., Metcalfe M.G., Spiropoulou C.F., Rollin P.E. (2010). Reduced virus replication, proinflammatory cytokine production, and delayed macrophage cell death in human pbmcs infected with the newly discovered bundibugyo ebolavirus relative to zaire ebolavirus. Virology.

[10] Sanchez A., Lukwiya M., Bausch D., Mahanty S., Sanchez A.J., Wagoner K.D., Rollin P.E. (2004). Analysis of human peripheral blood samples from fatal and nonfatal cases of Ebola (Sudan) hemorrhagic fever: Cellular responses, virus load, and nitric oxide levels. J. Virol..

[11] Wauquier N., Becquart P., Gasquet C., Leroy E.M. (2009). Immunoglobulin G in Ebola outbreak survivors, Gabon. Emerg. Infect. Dis..

[12] Sobarzo A., Ochayon D.E., Lutwama J.J., Balinandi S., Guttman O., Marks R.S., Kuehne A.I., Dye J.M., Yavelsky V., Lewis E.C. (2013). Persistent immune responses after ebola virus infection. N. Engl. J. Med..

[13] Lamunu M., Lutwama J.J., Kamugisha J., Opio A., Nambooze J., Ndayimirije N., Okware S. (2004). Containing a haemorrhagic fever epidemic: The ebola experience in uganda (October 2000–January 2001). Int. J. Infect. Dis..

[14] Sobarzo A., Groseth A., Dolnik O., Becker S., Lutwama J.J., Perelman E., Yavelsky V., Muhammad M., Kuehne A.I., Marks R.S. (2013). Profile and persistence of the virus-specific neutralizing humoral immune response in human survivors of sudan ebolavirus (gulu). J. Infect. Dis..

[15] Sobarzo A., Perelman E., Groseth A., Dolnik O., Becker S., Lutwama J.J., Dye J.M., Yavelsky V., Lobel L., Marks R.S. (2012). Profiling the native specific human humoral immune response to Sudan Ebola virus strain gulu by chemiluminescence enzyme-linked immunosorbent assay. Clin. Vaccine Immunol..

[16] Dias J.M., Kuehne A.I., Abelson D.M., Bale S., Wong A.C., Halfmann P., Muhammad M.A., Fusco M.L., Zak S.E., Kang E. (2011). A shared structural solution for neutralizing ebolaviruses. Nat. Struct. Mol. Biol..

[17] Kohavi R. (1995). A study of cross. Proceedings of the 14th International Joint Conference on Artifcial Intelligence (IJCAI).

[18] Paweska J.T., Burt F.J., Swanepoel R. (2005). Validation of igg-sandwich and igm-capture elisa for the detection of antibody to rift valley fever virus in humans. J. Virol. Methods.

[19] Swenson D.L., Wang D., Luo M., Warfield K.L., Woraratanadharm J., Holman D.H., Dong J.Y., Pratt W.D. (2008). Vaccine to confer to nonhuman primates complete protection against multistrain ebola and marburg virus infections. Clin. Vaccine Immunol..

[20] Cavelti-Weder C., Babians-Brunner A., Keller C., Stahel M.A., Kurz-Levin M., Zayed H., Solinger A.M., Mandrup-Poulsen T., Dinarello C.A., Donath M.Y. (2012). Effects of gevokizumab on glycemia and inflammatory markers in type 2 diabetes. Diabetes Care.

[21] Dye J.M., Herbert A.S., Kuehne A.I., Barth J.F., Muhammad M.A., Zak S.E., Ortiz R.A., Prugar L.I., Pratt W.D. (2012). Postexposure antibody prophylaxis protects nonhuman primates from filovirus disease. PNAS.

[22] Sullivan N.J., Martin J.E., Graham B.S., Nabel G.J. (2009). Correlates of protective immunity for Ebola vaccines: Implications for regulatory approval by the animal rule. Nat. Rev. Microbiol..

[23] Shedlock D.J., Aviles J., Talbott K.T., Wong G., Wu S.J., Villarreal D.O., Myles D.J., Croyle M.A., Yan J., Kobinger G.P. (2013). Induction of broad cytotoxic t cells by protective DNA vaccination against marburg and Ebola. Mol. Ther..

[24] Marzi A., Yoshida R., Miyamoto H., Ishijima M., Suzuki Y., Higuchi M., Matsuyama Y., Igarashi M., Nakayama E., Kuroda M. (2012). Protective efficacy of neutralizing monoclonal antibodies in a nonhuman primate model of Ebola hemorrhagic fever. PLoS One.

[25] Baize S., Leroy E.M., Georges-Courbot M.C., Capron M., Lansoud-Soukate J., Debre P., Fisher-Hoch S.P., McCormick J.B., Georges A.J. (1999). Defective humoral responses and extensive intravascular apoptosis are associated with fatal outcome in ebola virus-infected patients. Nat. Med..

[26] Wong G., Richardson J.S., Pillet S., Patel A., Qiu X., Alimonti J., Hogan J., Zhang Y., Takada A., Feldmann H. (2012). Immune parameters correlate with protection against ebola virus infection in rodents and nonhuman primates. Sci. Transl. Med..

[27] Wong G., Kobinger G.P., Qiu X. (2014). Characterization of host immune responses in ebola virus infections. Expert Rev. Clin. Immunol..

[28] Hoenen T., Groseth A., Feldmann H. (2012). Current Ebola vaccines. Expert Opin. Biol. Ther..

[29] Sullivan N.J., Hensley L., Asiedu C., Geisbert T.W., Stanley D., Johnson J., Honko A., Olinger G., Bailey M., Geisbert J.B. (2011). Cd8+ cellular immunity mediates rad5 vaccine protection against ebola virus infection of nonhuman primates. Nat. Med..

