# Supplementary material for: Immune Memory to Sudan Virus: Comparison between Two Separate Disease Outbreaks
Source: Viruses. 2015 Jan 6;7(1):37–51. doi: 10.3390/v7010037 (PMC4306827; doi:10.3390/v7010037)
Supplement: Supplementary File 1 [file viruses-07-00037-s001.pdf]

Supplementary Materials

Immune Memory to Sudan Virus: Comparison between Two Separate Disease Outbreaks

Ariel Sobarzo, Yael Eskira, Andrew S. Herbert, Ana I. Kuehne, Spencer W. Stonier, David E. Ochayon, Shlomit Fedida-Metula, Steven Balinandi, Yaara Kislev, Neta Tali, Eli C. Lewis, Julius Julian Lutwama, John M. Dye, Victoria Yavelsky and Leslie Lobel

Table S1. Subject profiles.

		Gender	Age	Ebola Infection in 2012	HIV
S-1	Kibaale, Uganda	F	36	+	-
S-2		F	35	+	-
S-3		F	30	+	-
S-4		F	61	+	-
S-5		F	29	+	-
N-1		F	30	-	-
N-2		F	36	-	-
N-3		M	42	-	+
N-4		F	37	-	-
N-5		M	27	-	-
S-1	Gulu, Uganda	F	58	+	-
S-2		F	44	+	-
S-3		F	20	+	-
S-4		F	32	+	-
S-5		F	56	+	-
S-6		M	42	+	-
N-1		M	25	-	-
N-2		M	32	-	-
N-3		F	27	-	-
N-4		F	37	-	-

S—Ebola survivors, N—Non-infected control. M-male, F-female.

Table S2. Survivor's hospitalization profiles.

No.		Hospital Admission	Hospitalization Duration (Days)	Treatment During Hospitalization †	Treatment After Release	Post Infection Symptoms &
S-1	*Kibaale*	Kagadi	10	-	-	+
S-2		Kagadi	30	-	-	+
S-3		-	-	-	-	+
S-4		-	-	-	-	+
S-5		-	-	-	-	+
S-1	*Gulu*	Gulu	14	-	-	+
S-2		Lacor	30	-	-	+
S-3		Lacor	30	-	-	+
S-4		Gulu	14	-	-	+
S-5		Gulu	14	-	-	+
S-6		Lacor	10	-	-	+

† All survivors received standard supportive care, including nutrition, electrolytes and liquids. No specific treatment against EHF was provided. & All survivors reported some post-infection symptoms associated with EHF recovery. Symptoms included impaired vision, muscle and joint pain, weakness and appetite loss. S—Ebola survivors. "-" Negative treatment or hospitalization, "+"—Positive treatment.

© 2015 by the authors; licensee MDPI, Basel, Switzerland. This article is an open access article distributed under the terms and conditions of the Creative Commons Attribution license (http://creativecommons.org/licenses/by/4.0/).
